# First report of *Kingella kingae* diagnosed in pediatric bone and joint infections in Morocco

**DOI:** 10.1186/s12879-021-06361-8

**Published:** 2021-07-20

**Authors:** Kaoutar Moutaouakkil, Bouchra Oumokhtar, Hicham Abdellaoui, Samira El Fakir, Btissam Arhoune, Mustapha Mahmoud, Karima Atarraf, Moulay Abderrahmane Afifi

**Affiliations:** 1 Laboratoire Pathologie Humaine Biomédecine et Environnement, Faculté de Médecine et de Pharmacie de Fès (FMPF), Université Sidi Mohammed Ben Abdellah (USMBA), Fès, Morocco; 2 Service de traumato-orthopédie pédiatrique, CHU Hassan II. Laboratoire Pathologie humaine Biomédecine et Environnement. Faculté de Médecine et de Pharmacie, Université Sidi Mohamemd Ben Abdellah, Fès, Morocco; 3 Laboratoire Epidémiologie, Recherche Clinique et Santé Communautaire. Faculté de Médecine et de Pharmacie, Université Sidi Mohammed Ben Abdellah, Fès, Morocco; 4 Laboratoire Centrale d´analyses médicales, CHU Hassan II. Faculté de Médecine et de Pharmacie, Université Sidi Mohammed Ben Abdellah, Fès, Morocco

**Keywords:** *Kingella kingae*, Bone and joint infection, Negative culture, Morocco

## Abstract

**Background:**

The progress of diagnostic strategies and molecular methods improved the detection of *Kingella kingae* in bone and joint infections, and now, *Kingella kingae* is being increasingly recognized as the most frequent cause of bone and joint infection BJI in early childhood. The main objective of this prospective study is to report the frequency of *Kingella Kingae* in negative culture bone and joint pediatric infections, and to describe the clinical and biologic features of these children.

**Methods:**

From December 2016 to June 2019, we selected all hospitalized patients with suspected BJI. When culture was negative on the fifth day, children under 10 years were subsequently included in the study, and PCR assay was performed systematically for researching *K. kingae* specific gene *cpn60*. Microbial culture and identification were made using standard bacteriological methods. The demographics, clinical, laboratory, radiographic and clinical features were reviewed from medical records.

**Results:**

We enrolled 65 children with culture negative BJI, 46 of them having under 10 years old have been screened for the *cpn60* gene. Thus, the gene encoding *Kingella kingae* was positive for 27 BJI cases (58.7%). The mean age of children was 3.02 years, 55.6% were aged 6 months-4 years and 29.6% of them were aged 5–10 years. The male to female ratio was 1.7 and 16 cases (59.26%) occurred during the fall-winter period. The most frequent BJI type was septic arthritis (77.8%) and the most affected sites were knee (51.9%) and hip (37.0%). We recorded a mild clinical picture with normal to mildly raised inflammatory markers. All patients had good clinical and functional outcomes, with no serious orthopedic sequelae..

**Conclusion:**

*K kingae* is an important pathogen of culture-negative BJI in Moroccan children. PCR testing should be performed in culture-negative cases of children not only in the typical age range of 6 months to 4 years. When implemented in the routine clinical microbiology laboratory, a specific *K. kingae* PCR assay can provide a better diagnostic performance of BJI.

## Background

Bone and joint infections (BJI) in children present a significant clinical challenge that could lead to life-long disability and death if not treated urgently [1]. Until recently, *Staphylococcus aureus* was considered the most common microorganism responsible for these infections. However, the progress of molecular diagnostic methods improved the detection of *Kingella kingae* from bone and joint samples. Currently, this bacteria is recognized in several countries, as the most frequent cause of BJIs especially among children aged between 6 and 48 months old. Affected children often have few signs and symptoms, and generally, clinical, radiological manifestations and biological inflammatory response to *K. kingae* BJI are mild to moderate [2].

*K. kingae* is typically a frequent component of the oropharyngeal microbiota of healthy young children. This asymptomatic colonization facilitates transmission between children notably through respiratory secretions [2]. Thus, the colonized epithelium is the gateway for the bacterium into the bloodstream where it disseminates to distant sites including bones, joints or endocardium. Ceroni et al. have reported a great risk of BJI with oropharyngeal carriage of *K. kingae* notably in children < 4 years of age, corresponding to the period of maximal oropharyngeal colonization. After 4 years, the carry rate decreases significantly [3].

Using culture-based diagnostic methods in our hospital, we frequently observe negative cultures in pediatric BJI, and actually, we have no local data on the involvement of K. K in this infection type. It was reported that *K. kingae* represented 30.8% (711/2308) of pediatric cases with culture and/or PCR proven musculoskeletal infections [4]. Recently, improvement of nucleic acid amplification assay targeting either the *rtx* operon or the *groEL* gene (also known as *cpn60*) allowed a further increase in the diagnostic capability for pediatric *K. kingae* BJI [5].

*K. kingae* infections have been reported mainly in the countries of the developed world whereas reports from the developing world are still scarce, owing to the high charge of PCR technologies required to detect the bacteria [6]. In Morocco and to the best of our knowledge, no studies have been published on *K. kingae* BJI. Therefore, the objective of this study was to investigate the frequency of pediatric *K. Kingae* BJI diagnosed in negative culture by PCR, and also to describe the clinical and biologic features of patients.

## Methods

### Study design and setting

From December 2016 to June 2019, we selected all patients with suspected BJI hospitalized in Pediatric Orthopedic Traumatology Ward (Hassan II UH, Fez). This surgical department has 28 beds, providing medical-surgical treatment for pediatric orthopedic disorders and also diseases of the musculoskeletal system. It’s the only tertiary care facility of its kind in Fez, a city with an estimated population of approximately 1.5 million inhabitants.

Patients were subsequently included in the study when the culture was negative on the fifth day, and we performed PCR assay systematically for researching *K. kingae* specific gene. Two types of infection were distinguished: septic arthritis and bone infections, including osteitis and osteomyelitis. We excluded patients above 10 years old and those with osteosynthesis material.

This study was approved by local Joint Research Ethics Committee. Informed consent was obtained from each parent’s patient before inclusion.

### Sampling and bacterial analysis

Blood samples, joint fluid or bone aspirate were shipped to the laboratory immediately after sampling. Each specimen was divided into two parts; the first one was inoculated into the rich medias: chocolate agar (PVX, BioMerieux®) and blood agar (COS, BioMerieux®) and then incubated at 37 °C for 24 h in aerobic and anaerobic conditions. The second one was inoculated into blood culture bottles (BD BACTEC®) and incubated using a BD BACTEC™ instrumented blood culture system for 10 days. Isolates were identified using standard methods and confirmed by Api System (BioMerieux®, Marcy l’Etoile, France).

### Molecular analysis

*K kingae* PCR testing was performed using residual bacterial culture-negative samples. These residual samples were stored at − 80 °C until used for DNA extraction. The total DNA was extracted from specimens by using the PrepMan™ Ultra DNA extraction kit (Applied biosystems®) according to the instruction. Then, the DNA concentration was measured by a NanoDrop 1000 spectrophotometer.

The DNA extracted were screened by PCR assay protocol targets the primers shown to be specific for the *cpn60* gene (also known as *gro*EL), which encode the Cpn60 chaperone protein: *KkF* (5′ – CTT-GCG-AAA-CAT-ACG-AGC-AA – 3′) and *KkR* (5′ – CCA-AAC-CAG-CGA-TTT-GTT-TT – 3′). The amplification conditions were described previously [7].

DNA *K. kingae* positive control was given graciously by Pr. Philippe Lanotte (CHRU de Tours). PCR products were detected on 1% agarose gel (FMC Bioproduct, Rockland, ME) after ethidium bromide staining, UV illumination and photographed by an Olympus digital camera (Olympus Soft Imaging Solutions GmbH, Münster, Germany).

### Data analysis

The socio-demographic characteristics of patients were collected prospectively using a standard written questionnaire. Clinical data were recorded from the medical record of each patient, and the following data were collected: clinical and laboratory parameters at presentation; imaging studies; surgical procedures; duration of hospitalization; antibiotic treatment; complication and sequelae. The biologic evaluation comprised peripheral white cell counts (WBC), C-reactive protein levels (CRP), and erythrocyte sedimentation rate (ESR).

Three categories of patients have been defined. The first group of young infants under 6 months corresponds to the end of maternally derived immunity. The second group of older infants comprises children from six to 48 months, where *K kingae* is recognized as the primarily affecting agent of this age. The third group includes juveniles over 4 years old and up to 10 years.

Results for quantitative variables were presented as mean ± standard deviation and for qualitative variables as number (percentage). Identification of potential risk factors associated with the infection by *K. kingae* was performed as appropriate. Chi-square test and Fisher’s exact test were used to establish a significant association between patients with *K. kingae* BJI and those with no *k. kingae* diagnosed. The *P <* 0.05 was deemed as statistically significant. Data were analyzed using SPSS software version 20 (IBM Corporation, Chicago, IL, USA).

## Results

We recorded 110 children diagnosed with BJI, 72 boys and 38 girls with ages ranging from 20 days to 15 years old. Bacterial culture from the joint, pus samples or bone tissue proved positive in 40.9% (45/110) among which 53.3% (24/45) were from septic arthritis, 31.1% (14/45) and 15.6% (7/45) respectively from osteomyelitis and multifocal abscess. *Staphylococcus aureus* methicillin-sensitive (except one isolate) was the most common causative organism identified (82.2%, 37 of 45). However, *K. kingae* was not revealed by the blood culture system despite the majority of BJI cases had not received antibiotics before surgical specimen.

Moreover, we showed that 59.1% of cases (65 of 110) were culture negative. Among them, the most frequent BJI types were septic arthritis (75.4%) diagnosed following standard criteria adopted routinely in the ward, notably temperature above than 38.3 °C and pain in the suspected joint that which got worse with notion, swelling in the suspected joint or systemic symptoms. Next comes osteomyelitis (18.5%), and spondylodiscitis (3.1%). The most frequent sites of infection were knee and hip by 45.7 and 34.8% respectively.

For this study we selected only patients having under 10 years old (*n* = 46). The mean age of children managed was 4.55 ± 3.11 years, 29 (63.0%) were boys and almost one-third of the patients were from rural settings. The mean temperature at admission was 38.53 °C, The mean of CRP value was 94.78 mg/L, and the mean ESR was 55.33 ± 32.52 mm/H.

All cultures were negative despite inoculating blood culture vials with joint fluid samples. However, 37 samples were positive for *cpn60*, the gene encoding *K. kingae* detected by PCR (58.7%). The mean age of *K. kingae* positive patients was 3.02 ± 2.50 years; ranging from < 1 month to 8 years. The male to female ratio was 1.7. Analyzing our results by age group showed that 14.8% (4/27) were under 6 months, and 2 of them were neonates, 55.6% of children were aged 6 to 48 months and 29.6% of infants were 48–96 months. *K. kingae* prevalence rate differs significantly between the population age groups (*P =* 0.009; Table 1). Out of 27 *K. kingae* BJI, 16 (59.26%) occurred during the fall-winter period. The time elapsed between the onset of symptoms and hospitalization was 10.4 days (SD 18.25 days).

**Table 1:** Data of patients with negative and positive *K. kingae* BJI cases

Characteristics	***K. kingae***- negative cases ***N*** = 19	***K. kingae***-positive cases ***N*** = 27 (58.7%)	***P-value***
**Demographics:**			
Age (years)			
< 6 months	0 (0)	4 (14.8)	0.009
6–59 months	5 (26.3)	15 (55.6)	
5 y-10 y	14 (73.7)	8 (29.6)	
Gender			
Male	12 (63.2)	17 (63.0)	0.618
Female	7 (36.8)	10 (37.0)	
Origin			
Rural	8 (42.1)	8 (29.6)	0.287
Urban	11 (57.9)	19 (70.4)	
**Admission features:**			
Type of the infection			
Septic arthritis	15 (78.9)	21 (77.8)	0.417
Osteomyelitis	3 (15.8)	4 (14.8)	
Multifocal abscesses	1 (5.3)	0 (0)	
Spondilodiscitis	0 (0)	2 (7.4)	
Site of the infection			
Single	17 (89.5)	24 (88.9)	0.448
Multiple	2 (10.5)	3 (11.1)	
Location of limb infection			
Upper	1 (5.3)	3 (11.1)	0.632
Lower	18 (94.7)	24 (88.9)	
Limb affected			
Hip	6 (31.6)	10 (37.0)	0.475
Femur	2 (10.5)	3 (11.1)	0.667
Knee	7 (36.8)	14 (51.9)	0.241
**Diagnosis on admission**			
Magnetic resonance imaging	1 (5.3)	2 (7.4)	0.632
Pathologic ultrasound	8 (42.1)	14 (51.9)	0.191
Joint effusion	5 (31.3)	5 (26.3)	0.519
Soft tissue infiltration	5 (31.3)	11 (57.9)	0.108
soft tissue abscesses	1 (6.3)	6 (31.6)	0.062
Abnormal plain radiograph	3 (15.8)	1 (3.7)	0.208
Purulent discharge	6 (31.6)	3 (11.1)	0.085

In addition, 77.8% of the *K. kingae*-positive children group had septic arthritis, 14.8% had osteomyelitis and 7.4% had spondylodiscitis. More than half had an infection of the knee joint, 37.0% of the hip, and 11.1% of the femur. No infection was shown in the shoulder, humerus, forearm, wrist and foot (Table 1).

Generally, we recorded a mild clinical picture with normal to mildly raised inflammatory markers. The median temperature reported at admission was 38.60 °C and the mean temperature was 38.62 °C. The mean CRP value was 96.31 mg/L, 85.2% of patients having values > 20 mg/L and 77.8% > 50 mg/L. The mean Erythrocyte sedimentation rate was 54.50 ± 35.65 (mm/H); and the mean WBC rate was 13,429.9 ± 6063.9 /mm^3^. Clinical features of the children with negative and positive *K. kingae* BJI are summarized in Table 2.

**Table 2:** Clinical features of patients with negative and positive *K. kingae* BJI cases

Clinical features on admission	***K. kingae***- negative cases ***N*** = 19	***K. kingae***-positive cases ***N*** = 27 (58.7%)	***P-value***
Fever (≥ 38) °C	12 (63.2)	19 (70.4)	0.421
Initial value, mean (±SD)	38.73 ± 0.64	38.62 ± 0.53	0.625
CRP (mg/l)			
Initial value, mean (±SD)	92.61 ± 79.95	96.31 ± 57.07	0.855
Elevated CRP on admission	17 (89.5)	23 (85.2)	0.516
ESR (mm/H)			
Initial value, mean (±SD)	56.44 ± 29.90	54.50 ± 35.65	0.896
Elevated ESR on admission	8 (42.1)	12 (44.4)	0.484
WBC /mm^3^			
Initial value, mean (±SD)	13,554.7 ± 4596.6	13,429.9 ± 6063.9	0.940
Elevated WBC on admission	15 (78.9)	21 (77.8)	0.925
Arthrotomy	8 (42.1)	19 (70.4)	0.053
Hospital stay, days, mean (±SD)	9.64 ± 5.80	11.17 ± 7.27	0.508

Two of the children found to be PCR-positive for *K. kingae* have prior antibiotic therapy before collection of joint fluid samples for culture. The average hospital stay was 11.17 days. Only one patient with *K. kingae* positive tibial acute osteomyelitis had a complication, consisting of metaphyseal pus collection that necessitates multiples drainages. This patient had evolution to chronic osteitis with a lesion of the proximal tibial growth plate. We reported good clinical and functional outcomes following antibiotic treatment for all the rest patients.

## Discussion

Bone and joint infection BJI is a potentially devastating disorder with a high incidence of severe and long-lasting sequelae, particularly in growing children. Improving patient management requires determining the causative agent to prevent the risk of long-term disabilities [8]. *Staphylococcus aureus* is the most common pathogen in acute BJI, being identified in 70 to 90% of culture positive cases. In our study, *Staphylococcus aureus* was the main causative agent of infections recovered at 82.2% on a positive culture. However, we showed that 59.1% of samples (65 of 110) remained culture negative. Dodwell has reported that no organism is identified in up to 55% of pediatric BJI cases, even if appropriate samples had been obtained [9].

Recently, molecular diagnosis has significantly increased positive results, especially among *K. kingae* infections. In countries where *K. kingae*-specific real-time PCR (RT-PCR) assays are routinely employed this organism is recognized as the main agent of BJI in young children [10, 11]. In fact, to date, little is known on the epidemiology of *K. kingae* BJIs in children in the African continent. To the best of our knowledge, we report the first study of *K. kingae* BJI among Moroccan children population. We revealed that *K. kingae* was the leading cause (58.7%) of culture-negative BJIs in young children. This rate are higher than 36.07 and 45% reported in France [10, 12], or 25.93% in Ottawa [13]. *K. kingae* infections seem to be closely correlated to children’s ages. In a meta-analysis, Wong et al. reported 47.6% of patients under 48 months of age were diagnosed with *K. kingae* BJI infections [4]. In this study, 55.6% of children were aged 6 to 48 months, and 14.8% (4/27) were under 6 months, 2 of them were aged < 1 month, with no history of trauma or unusual infections, and no warning signs of immunosuppression were found. Also, 29.6% (8/27) of children were over 48 months (5 years and older (5–8.2 years), close contact with younger children or siblings is very likely, which may explain this results. This finding is not corroborating with the classic representation of *K. kingae* BJI, which occurs in children between 6 months and 4 years old. Shahrestani et al. [14] and more recently, Ceroni et al. have established the possibility of *K. kingae* osteoarticular infections in older immunocompetent children [15]. Also, we diagnosed a large proportion of *K. kingae* patients during the fall to winter season. Wong et al. have reported that this seasonal variation is likely associated with respiratory viral infections and stomatitis, allowing for passage of the colonizing agent through the breached epithelium [4]. Concerning the site of infection, *K. kingae* are responsible of septic arthritis with variable of 45 to 69% [8, 10, 12, 16]. We recorded 21 cases of septic arthritis (77.8%), 4 osteomyelitis (14.8%) and 2 spondylodiscitis. Similarly to published data, our finding confirms that infection tends to affect the lower extremity the most, and the knee is often involved [16], and almost all cases present a moderate clinical picture with normal to slightly elevated inflammatory markers [6]. in fact, chronic osteomyelitis and orthopedic sequelae seem to be uncommon in *K. kingae* BJI in young children [2, 8].

Unfortunately, we were unable to establish an antimicrobial susceptibility pattern, because all culture was negative despite inoculation into blood culture vials. It has been recognized that *K. kingae* is most often sensitive to many classes of antibiotics (beta-lactams, macrolides, aminoglycosides, fluoroquinolones, tetracyclins) [13]. For managing BJI in our ward, empirical antibiotic therapy is started with intravenous antibiotics (clavulanic-acid/amoxicillin and gentamycin), and after discharge, treatment continues orally by 10 days to 3 weeks according to the judgment of the attending physician. In the case of documented *K. kingae* BJI, the antibiotic treatment consisted of oral clavulanic-acid/amoxicillin for 14 to 21 days. Several authors have suggested switching to trimethoprim-sulfamethoxazole once *K kingae* infection was identified [6, 14].

For PCR analysis, we targeted the *groEL* gene (also known *cpn60)*, a housekeeping gene encoding a chaperone protein recognized as a universal bacterial marker [17]. Recently, El Houmani et al. provided evidence that *kkgroEL* gene could discriminate even *K. kingae* from *K. negevensis* [14].

Our study had several limits, the first of which was the one site of study and the small sample size. Despite this, we recorded 58.7% *K. kingae* BJI, providing evidence that *K. kingae* is circulating in our region as well. Also, we could not define antimicrobial susceptibility patterns in our region because no *K. Kingae* was growing in culture media even we inoculated joint samples into aerobic blood vials. Further studies are required to determine the prevalence of *K. kingae* carriage and BJI in other Moroccan sites.

## Conclusion

*K kingae* is an important pathogen of culture-negative BJI in Moroccan children. PCR testing should be performed in culture-negative cases of children not only in the typical age range of 6 months to 4 years. When implemented in the routine clinical microbiology laboratory, a specific *K. kingae* PCR assay can provide a better diagnostic performance of BJI.

## Data Availability

The datasets used and/or analyzed during the current study are available from the corresponding author on reasonable request.
